# Pediatric cystinosis: Corneal cystine deposits and papilledema in a 4-year-old:  A case report

**DOI:** 10.3892/mi.2025.242

**Published:** 2025-05-14

**Authors:** Dharamveer Singh Choudhary, Jeba Shaheen, Ritu Kala, Ajay Dhakad, Bhuvanesh Sukhlal Kalal

**Affiliations:** 1 Department of Ophthalmology, Swai Man Singh Medical College and Hospitals, Jaipur, Rajasthan 302004, India; 2 Department of Biotechnology, Swai Man Singh Medical College and Hospitals, Jaipur, Rajasthan 302004, India; 3 Department of Pharmacology and Nutritional Sciences, College of Medicine, University of Kentucky, Lexington, KY 40536, USA

**Keywords:** corneal cystine crystals, ocular manifestations, papilledema, cysteamine therapy, intracranial pressure

## Abstract

Cystinosis is a rare autosomal recessive lysosomal storage disorder characterized by the accumulation of cystine within lysosomes, leading to multi-systemic complications. The present study describes the case details (the presentation and management) of a 4-year-old female child diagnosed with infantile cystinosis, further complicated by distal renal tubular acidosis and stage 4 chronic kidney disease. The patient exhibited significant ocular manifestations, notably bilateral corneal cystine crystal deposits, observed as a shimmering effect under slit-lamp biomicroscopy and marked papilledema in both eyes. Fundoscopic examination also revealed retinal cystine deposits, indicating systemic involvement. The systemic complications included renal dysfunction requiring ongoing dialysis and bicarbonate supplementation to manage metabolic acidosis, as well as elevated intracranial pressure. Ophthalmological management focused on vision preservation through corrective lenses and topical cysteamine eye drops to reduce corneal cystine accumulation. Regular follow-up appointments were scheduled to monitor corneal clarity and optic nerve health. The case described herein underscores the complexity of cystinosis and the critical need for a multidisciplinary approach involving ophthalmology, nephrology, and neurology. Early diagnosis and timely therapeutic interventions are essential to mitigate the progressive nature of the disease and improve patient outcomes. The present case report also highlights the challenges in managing the condition, including treatment adherence and potential complications, and emphasizes the importance of continued research to develop more effective therapies and improve the quality of life for affected individuals.

## Introduction

Cystinosis is a very rare autosomal recessive lysosomal storage disorder that leads to the buildup of cystine in the lysosomes of affected cells (1). There are three forms of the disease, with the infantile form typically being the most severe, as it presents both ocular and systemic symptoms at an early stage (2). This form is associated with severe or truncating mutations on both alleles, and adult forms usually involve at least one milder mutation (3). Infantile cystinosis, the most severe and common form, presents within the first year of life with Fanconi syndrome, growth retardation and progressive renal failure (4).

Cystinosis often leads to systemic issues, such as renal failure and eye conditions, particularly corneal cystine deposits (1). The underlying cause is the impaired transport of cystine out of lysosomes due to mutations in the *CTNS* gene, which encodes cystinosin, a transporter for lysosomal cysteine (5). This accumulation results in cellular dysfunction and tissue damage, leading to the systemic and ocular symptoms observed in cystinosis. The disorder follows an autosomal recessive inheritance pattern, affecting ~1 in 100,000 to 200,000 live births (6).

There are three clinical subtypes of cystinosis: Infantile (nephropathic), juvenile (intermediate) and adult (non-nephropathic or ocular) (7). Infantile cystinosis, the most severe and common form, presents within the first year of life with Fanconi syndrome, growth retardation and progressive renal failure (1). Without treatment, affected individuals typically develop end-stage renal disease by adolescence. The juvenile form manifests later in childhood with milder renal impairment, while the adult form primarily involves ocular symptoms, such as photophobia due to corneal cystine crystal deposition.

Ophthalmic complications in cystinosis are significant and include corneal cystine deposits, photophobia, conjunctival damage, retinal degeneration, and, in rare cases, optic nerve involvement (8). Papilledema is an unusual finding and may indicate increased intracranial pressure (ICP), which can be secondary to chronic kidney disease (CKD), electrolyte disturbances, or other systemic factors. Early diagnosis through biochemical testing (leukocyte cystine levels) and genetic screening is crucial for initiating timely treatment (9).

Despite therapeutic advancements, managing cystinosis remains challenging due to medication adherence issues, potential side-effects of cysteamine and the progressive nature of the disease. Given its multi-systemic impact, a multidisciplinary approach involving nephrologists, ophthalmologists, neurologists and geneticists is essential for optimizing patient outcomes. The present study describes the case of a patient with infantile cystinosis that exhibited both ocular and systemic symptoms. The aim of the present study was to underscore the diagnostic and therapeutic challenges this condition presents and to highlight the necessity of a multidisciplinary approach to improve the quality of life and long-term outcomes of patients.

## Case report

### Patient history

A 4-year-old girl presented to the Department of Ophthalmology, Swai Man Singh Medical College and Hospitals, Jaipur, India with a primary concern of visual disturbances in both eyes. Her parents had observed a gradual increase in her difficulty with visual tasks, such as recognizing familiar objects and moving around her environment. She had a medical history notable for cystinosis, which was diagnosed when she was 18 months old, leading to distal renal tubular acidosis (dRTA) and stage 4 CKD. The patient was receiving regular dialysis and was administered bicarbonate supplements to manage her renal acidosis. There was no record of eye injuries, previous surgeries, or other notable illnesses. Despite her ongoing health challenges, her developmental milestones were appropriate for her age.

### Ocular examination

**Figure 1:**
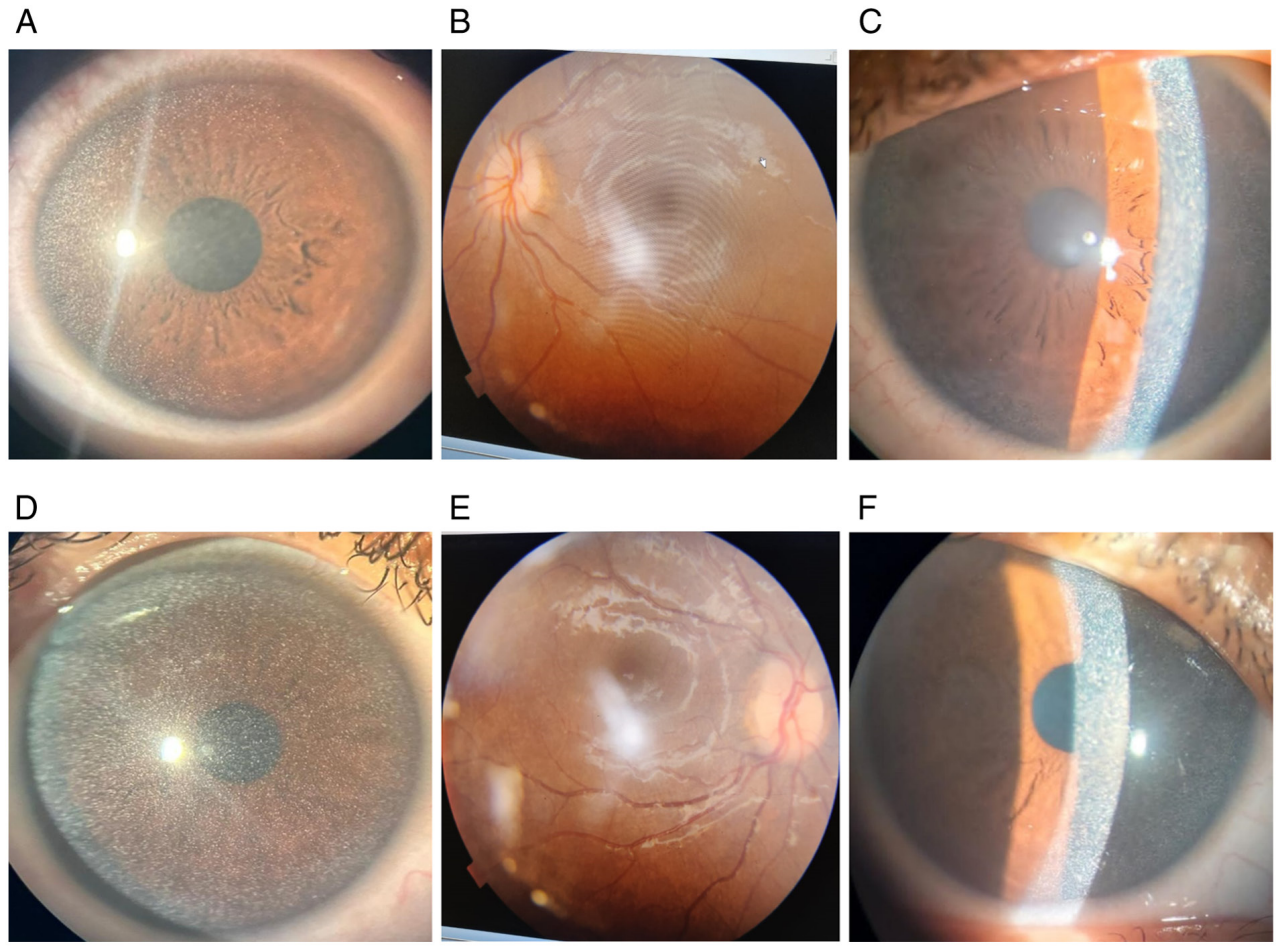
Corneal involvement in pediatric cystinosis. Diffuse light examination of the right (A) and left (D) eye indicates fine homogenous cystine crystal deposits across the cornea from limbus to limbus. Fundus of right (B) and left (E) eye indicating fine cysteine deposits. A slit-lamp photograph of the right (C) and left (F) eye indicates dense cystine crystal deposits within the corneal stroma, sparing the epithelium and endothelium.

The best-corrected visual acuity (BCVA) in both eyes was finger count at six feet, indicating severe visual impairment. Refraction revealed hyperopic prescriptions of +2.50 diopters in the right eye and +2.50 diopters with +0.50 diopters at 90˚ in the left eye. An anterior segment examination revealed diffuse bilateral corneal cystine crystal deposits (Fig. 1A and D), which created a characteristic shimmering effect under slit-lamp biomicroscopy. A fundoscopic examination indicated marked papilledema in both eyes, consistent with elevated intracranial pressure (Fig. 1B and E). Additionally, bilateral retinal cystine deposits were observed, further supporting systemic involvement (Fig. 1C and F). Optic nerve head swelling, confirmed by B-scan ultrasonography, revealed optic disc diameters of 3.8 mm in the right eye and 4.1 mm in the left eye, with notable optic disc elevation.

### Systemic associations

**Figure 2:**
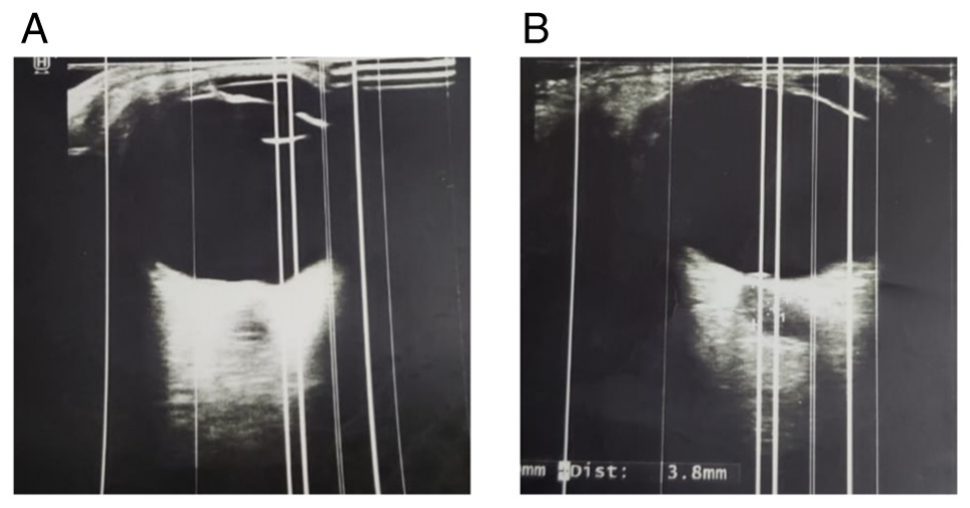
Optical coherence tomography images of intraocular status of (A) left and (B) right eye.

The systemic condition of the patient was primarily affected by complications arising from cystinosis. Renal function tests (urea, 92.57 mg/dl; creatinine, 1.46 mg/dl; serum thyroid-stimulating-hormone, 10 mIU/l) indicated stage 4 CKD, which required ongoing dialysis and bicarbonate supplementation to manage persistent metabolic acidosis. A neurological evaluation indicated elevated intracranial pressure, likely resulting from a combination of CKD and electrolyte imbalances (Fig. 2). A neurological examination revealed no motor or sensory deficits, and a referral was made to a pediatric neurologist for further evaluation and management of the intracranial hypertension.

### Diagnosis

The patient was diagnosed with corneal cystine deposits due to systemic cystinosis and papilledema linked to elevated intracranial pressure, likely stemming from CKD and its associated complications. The ocular findings were directly tied to the systemic condition, underscoring the interconnected nature of her health issues.

### Ophthalmological care

The main objective of ophthalmological management was to protect the remaining vision of the patient and relieve symptoms. Corrective lenses were prescribed to enhance her visual acuity. Topical cysteamine eye drops were commenced as a standard treatment to decrease the accumulation of corneal cystine crystals, which would help improve corneal clarity and prevent further visual decline. Artificial tears were suggested to alleviate dryness on the ocular surface and improve patient comfort. Regular follow-up appointments were arranged to track the progression of corneal deposits and papilledema, with slit-lamp and fundoscopic examinations conducted at each visit.

### Systemic treatment

The management of the systemic condition of the patient focused on controlling the complications associated with cystinosis and CKD. Dialysis was continued, and bicarbonate supplementation was provided to manage metabolic acidosis. The patient was referred to a pediatric neurologist to investigate the cause of elevated intracranial pressure and to commence appropriate treatment. This included monitoring for any signs of worsening papilledema and neurological symptoms that may necessitate cerebrospinal fluid pressure management or other interventions. Nutritional support and growth monitoring were also prioritized to ensure the overall development and health of the patient.

### Follow-up protocol

A thorough follow-up protocol was established, which included corneal evaluations every 3 to 6 months and fundoscopic examinations every 1 to 2 months. This approach aimed to ensure the early detection of any deterioration in corneal clarity or optic nerve health. Effective coordination among the ophthalmology, nephrology and neurology teams was essential for providing comprehensive care.

## Discussion

The case presented herein illustrates the complex effects of cystinosis on pediatric patients, particularly as regards its ocular and systemic manifestations. Corneal cystine deposits, a defining feature of the disease, arise from the accumulation of cystine in lysosomes and can severely affect vision (1). These deposits can worsen over time and may lead to corneal scarring, if left untreated. Topical cysteamine has proven effective in reducing corneal crystals; however, maintaining adherence to treatment can be difficult, particularly for young children (10-12).

Papilledema, as observed in the patient in the present study, is a rare, yet serious complication of cystinosis. It can occur due to elevated intracranial pressure resulting from chronic kidney disease, electrolyte imbalances, or other systemic issues. If left untreated, papilledema can result in optic atrophy and permanent vision loss (13). Although a lumbar puncture was not performed, the patient's clinical presentation and systemic findings were consistent with secondary ICP elevation. The link between CKD and papilledema in patients with cystinosis has been well-documented; however, it remains multifactorial (3,14). In the patient in the present study, electrolyte imbalances (hypokalemia and metabolic acidosis), fluid retention and possible hypertension could have contributed to the increased ICP. At the same time, the multidisciplinary approach taken in the present case was instrumental in managing the condition of the patient (8,15,16). Additional neuroimaging and cerebrospinal fluid analysis could have helped confirm the diagnosis and rule out other contributing factors. The pathophysiology of ICP regulation in cystinosis remains poorly understood, warranting further investigations. Improved neuro-ophthalmologic screening guidelines may facilitate earlier detection and intervention in such cases.

Cysteamine therapy is effective in delaying disease progression; however, it requires strict adherence. Frequent dosing (4-6 times daily) remains challenging, particularly in pediatric patients (17). Research has indicated that while cysteamine therapy significantly reduces corneal crystal density and improves photophobia, complete clearance is often not achieved (18). Moreover, adverse effects such as ocular irritation, burning sensation, and the potential for conjunctival hyperemia can further affect compliance. Beyond eye health, systemic cysteamine therapy can lead to several side-effects that should be considered when planning long-term treatment (19). Common issues include gastrointestinal symptoms, such as nausea, vomiting and abdominal pain, as well as halitosis, which is a result of dimethylsulfide production. Given these challenges, future research is required to explore new drug delivery systems, such as sustained-release formulations, that could reduce dosing frequency and minimize some side-effects. This could help improve both the effectiveness of the treatment and patient compliance. By better addressing these concerns, researchers can work toward developing more balanced, long-term strategies for managing cystinosis in children, ensuring that the benefits of treatment outweigh the challenges posed by side-effects (4).

The importance of genetic counseling and early diagnosis in managing cystinosis cannot be emphasized enough. Detecting the condition early enables the start of systemic cysteamine therapy, which can help attenuate the progression of renal and other systemic complications. Additionally, regular eye examinations from infancy are vital for identifying and treating ocular issues before they affect vision. Genetic testing was not performed in the case in the present study, which represents a limitation of the present study. Molecular testing of *CTNS* mutations is crucial for definitive diagnosis, prognosis estimation and family counseling. Future studies are thus required to integrate genetic testing into standard diagnostic workflows to enhance early detection and intervention. Newborn screening programs using tandem mass spectrometry to detect elevated cystine levels in leukocytes have been proposed in high-risk populations, but are not yet widely implemented (20). Expanding awareness and accessibility to genetic diagnostics may improve patient outcomes.

The long-term management of cystinosis requires coordinated follow-up across multiple specialties. Regular ophthalmologic evaluations, including corneal clarity assessments, optic nerve examinations and visual acuity monitoring, should be scheduled every 3-6 months. Monitoring for systemic complications, such as gastrointestinal side-effects, halitosis, bone abnormalities, particularly CKD progression and metabolic imbalances, is essential for timely interventions (21). Patients should also undergo periodic neuro-ophthalmologic evaluations to track ICP and visual function changes. Given the burden of lifelong therapy, quality-of-life assessments are crucial. Pediatric patients face challenges with medication adherence, social integration and developmental milestones (22). Psychological support and patient education programs should be integrated into care plans to improve adherence and mental well-being. Advances in cysteamine formulations, gene therapy and novel neuroprotective strategies hold promise for improving long-term outcomes in patients with cystinosis (23).

The present study has several limitations which should be mentioned. The reliance on a single case restricts the generalizability of the findings, emphasizing the need for larger patient cohorts to better characterize the association between cystinosis and papilledema. Additionally, the absence of genetic testing represents a limitation, as molecular confirmation of *CTNS* mutations would have strengthened diagnostic accuracy. Furthermore, cerebrospinal fluid analysis and neuroimaging were not performed, limiting the ability to fully investigate the underlying cause of papilledema. Future research should focus on expanding genetic screening databases, identifying biomarkers for early disease detection, and exploring new therapeutic strategies to improve long-term outcomes.

In conclusion, the present study described the case of a 4-year-old girl with cystinosis, dRTA and CKD4, highlighting the significant systemic and ocular effects of the disease. Key findings included corneal cystine deposits and papilledema, which were addressed through a collaborative approach. The combination of eye care, systemic treatment and neurological assessment was crucial in managing the symptoms of the patient and preventing further complications. Early diagnosis, prompt intervention and consistent follow-ups are critical for achieving the optimal outcomes for pediatric patients with cystinosis. The present case report highlights the importance of teamwork in managing rare and complex conditions.

## Data Availability

The data generated in the present study may be requested from the corresponding author.
